# Relaxin-2 in Cardiometabolic Diseases: Mechanisms of Action and Future Perspectives

**DOI:** 10.3389/fphys.2017.00599

**Published:** 2017-08-18

**Authors:** Sandra Feijóo-Bandín, Alana Aragón-Herrera, Diego Rodríguez-Penas, Manuel Portolés, Esther Roselló-Lletí, Miguel Rivera, José R. González-Juanatey, Francisca Lago

**Affiliations:** ^1^Cellular and Molecular Cardiology Research Unit, Institute of Biomedical Research and University Clinical Hospital Santiago de Compostela, Spain; ^2^Centro de Investigación Biomédica en Red de Enfermedades Cardiovasculares Madrid, Spain; ^3^Cardiocirculatory Unit, Health Research Institute of La Fe University Hospital Valencia, Spain

**Keywords:** cardiovascular diseases, relaxin-2, metabolism, inflammation, therapy, heart

## Abstract

Despite the great effort of the medical community during the last decades, cardiovascular diseases remain the leading cause of death worldwide, increasing their prevalence every year mainly due to our new way of life. In the last years, the study of new hormones implicated in the regulation of energy metabolism and inflammation has raised a great interest among the scientific community regarding their implications in the development of cardiometabolic diseases. In this review, we will summarize the main actions of relaxin, a pleiotropic hormone that was previously suggested to improve acute heart failure and that participates in both metabolism and inflammation regulation at cardiovascular level, and will discuss its potential as future therapeutic target to prevent/reduce cardiovascular diseases.

## Introduction

In the last decades, cardiovascular diseases (CVDs) have remained as the first cause of death worldwide, being their prevalence boosted every year mainly due to our new way of life, based on the increased intake of cheaper energy-dense food and a sedentary lifestyle (Pérez-Martínez et al., 2017; WHO | Cardiovascular diseases (CVDs), 2017). Hand in hand with this increase in the prevalence of CVDs goes the increase in obesity (WHO | Obesity Overweight, 2016), which not only is a risk factor for CVDs by itself, but also promotes the development of other CVDs comorbidities/risk factors, including hypertension, insulin resistance, dyslipidemia, type 2 diabetes mellitus (T2DM) or the increase in systemic inflammation (Tune et al., 2017). In particular, the combination of abdominal obesity, hypertension, hyperglycemia and dyslipidemia is known as metabolic syndrome (Matsuzawa et al., 2011; Wiernsperger, 2013), and due to the increasing evidences relating the presence of metabolic syndrome to the development of cardiovascular events such as myocardial infarction or stroke, this state is now termed cardiometabolic syndrome (Wiernsperger, 2013). The main therapeutic approach to treat the cardiometabolic syndrome is focused on restoring the metabolic disorder to a normal state through weight reduction and the prescription of drugs such as anti-diabetics, statins, anti-inflammatories or anti-hypertensives (Duprez and Toleuova, 2013; Ginsberg, 2013; Wiernsperger, 2013; Soare et al., 2014; Desouza et al., 2015). Unluckily, the therapeutic approaches available nowadays to treat the pathologies that define the cardiometabolic syndrome are not sufficient, since this syndrome alters different metabolic pathways, mainly those regarding glucose and lipid metabolism, and affects diverse organs/tissues, including the liver, the muscles or the fat tissue, and, moreover, each individual can show different metabolic abnormalities (Wiernsperger, 2013). Thus, there is an urge to understand the signaling pathways of the different contributors to the development of cardiometabolic diseases (CMDs) in an attempt to find new possible targets that with their therapeutic modulation could improve CMDs treatment and/or prevention.

In this line, in the last years the obesity and the adipose tissue have received considerable attention regarding their potential contribution to the development of CMDs. It is well established that the adipose tissue functions as an endocrine organ by secreting a number of proteins/hormones (adipokines) mainly implicated in the regulation of metabolism and in the control of the inflammatory response (Mancuso, 2016). Obesity induces an imbalance in the adipokine production in favor of pro-inflammatory adipokines and in detriment of anti-inflammatory adipokines, leading to a low grade of chronic inflammation that promotes both systemic metabolic dysfunction and CVDs (Nakamura et al., 2014; Molica et al., 2015). In fact, inflammation is nowadays recognized as a central player in the development of CVDs and its complications (Ruparelia et al., 2016), and the study of this kind of hormones that influence metabolism and inflammation and which have been shown to have effects at cardiovascular level (not only adipokines, but also other hormones, such as ghrelin, which is mainly produced by the stomach (Lilleness and Frishman, 2016), nesfatin-1, that is widely expressed in the body, including the brain and the heart (Feijóo-Bandín et al., 2015), or prokineticin, secreted by immune cells and reproductive organs, and expressed in heart and kidney apart from the adipose tissue Nebigil, 2017) has raised a great interest among the scientific community regarding their potential role in the development/prevention of CMDs (Ingelsson et al., 2008; Athyros et al., 2010; Gonzaga et al., 2014; Chiara et al., 2015; Prinz and Stengel, 2016; Colldén et al., 2017). Hence, the study of this kind of proteins/hormones that participate in the regulation of metabolism and/or inflammation can shed light in the understanding of how cardiometabolic diseases behave, and contribute to the developing of new therapeutic approaches.

Relaxin is a hormone that was first identified as a reproductive hormone implicated in vasoregulation during pregnancy and the softening of the tissues of the birth canal during delivery (Bani, 1997), but that has been recently suggested to participate in metabolism regulation and to exert protective effects at cardiovascular level. This review outlines the functions of relaxin as a new potential metabolic hormone with cardiovascular actions and discusses its potential as future therapeutic target to prevent/reduce CMDs.

## Relaxin

**Figure 1:**
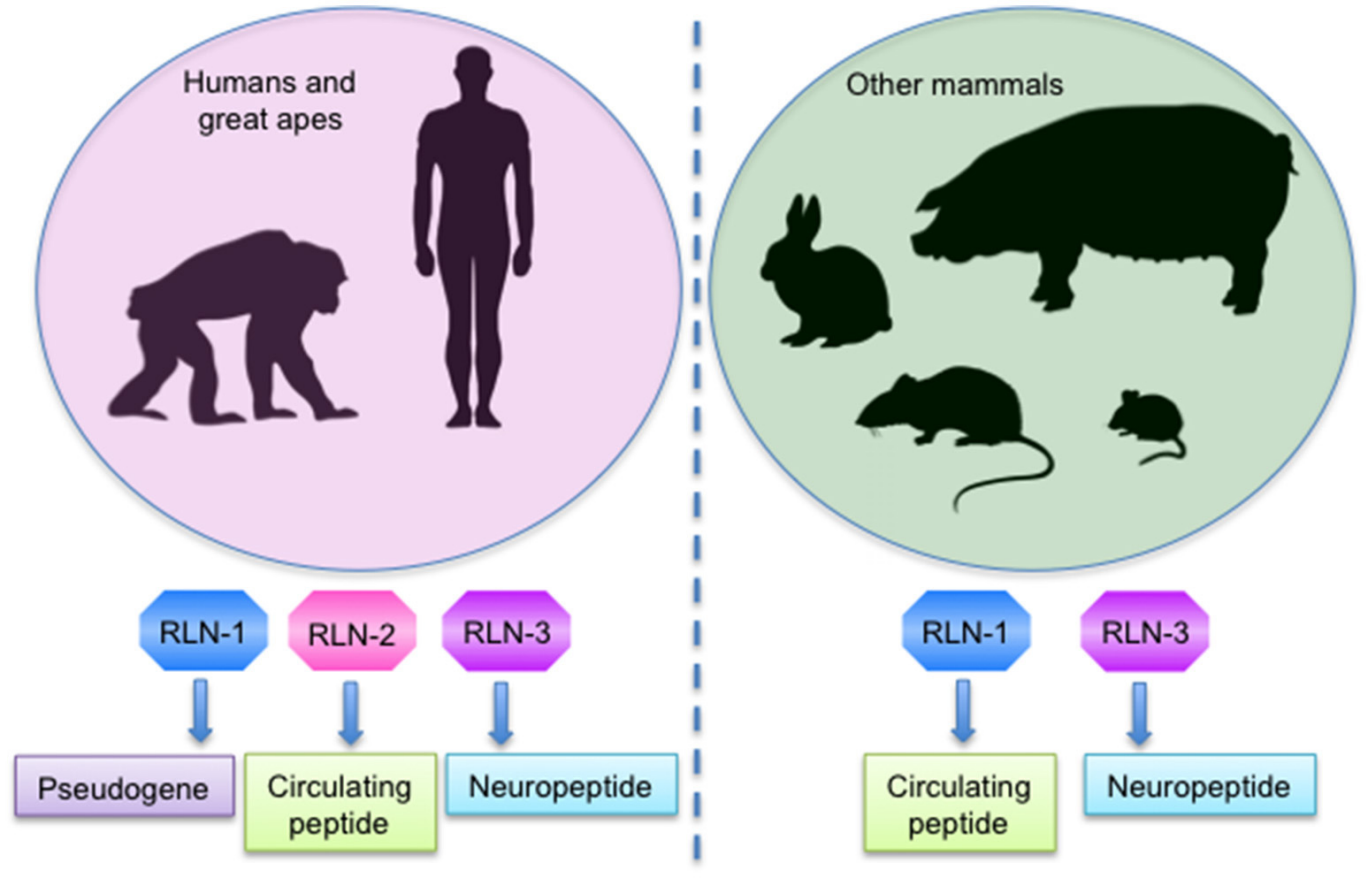
Expression of the mostly studied relaxin genes in mammals.

Relaxin is a 6 kDa hormone identified and named in 1926 by Frederick Hisaw due to its ability to induce the relaxation of the pubic ligaments and the softening of pubic symphysis just prior to delivery in pregnant gophers and guinea pigs (Hisaw, 1926; Wilkinson et al., 2005). Subsequently, different relaxin genes were discovered, so that nowadays the relaxin peptide family consists of seven peptides: relaxin (RLN)-1, RLN-2, RLN-3/insulin-like peptide (INSL)-7, and INSL3-6 (Bathgate et al., 2013). In humans and higher primates, there are three RLN genes: RLN-1, RLN-2, and RLN-3; however, the function of RLN-1 is unclear and it may even represent a pseudogene in these species. In contrast, other mammals have only the RLN-1 and RLN-3 genes. Importantly, the RLN-2 gene in humans and the RLN-1 gene from other mammals are equivalent and encode the relaxin peptides that circulate in blood during pregnancy, being RLN-2 in humans and great apes and RLN-1 in other non-primate species commonly referred to as relaxin (Wilkinson et al., 2005; Bathgate et al., 2013; in this review we will refer to RLX-2 and RLX-1 as relaxin), while the peptide encoded by the RLN-3 gene is a neuropeptide in all species (Figure 1; Dschietzig, 2014).

Although human relaxin was originally discovered as a hormone mainly secreted by the corpus luteum of the ovary that regulates the adaptive changes in pregnancy, it is also produced in non-pregnant women, and males also produce relaxin, being identified the presence of the relaxin peptide in prostate. Relaxin mRNA was also detected in other tissues, such as the endometrium, decidua, placenta, mammary gland, brain and the heart (Bathgate et al., 2013).

Initially, the different members of the relaxin family were discovered due to their similar structure and their roles in reproduction. However, nowadays we know that they participate in a wide range of physiological functions apart from reproduction, including stress, fear and anxiety responses, behavioral activation, mood, reward, depression, addiction, feeding behavior, metabolism, water drinking behavior, learning and emotional memory or somatosensory motor behavior (Gundlach et al., 2013). In some cases, they are expressed and have well conserved roles in different species, like RLX-3 and INSL-3, but in others, such as RLX-2, the expression and function differ between species (Bathgate et al., 2013).

### Relaxin activation and receptors

Relaxin is a two-chain peptide with a structure and processing similar to insulin. It is produced as a pro-hormone, containing a signal sequence and a B-C-A domain configuration, and after processing by prohormone convertases, the C domain is removed and three disulphide bonds are formed between six highly conserved cysteine residues in the A and B chains. Thus, the mature relaxin is constituted by the A and B chains with three disulphide bonds, like insulin (James et al., 1977; Bathgate et al., 2013). Human relaxin gene structure was first identified in 1983, showing a highly conserved sequence within the B-chain (R-X-X-X-R-X-X-I/V-X) that was later found to be indispensable for the binding to relaxin receptors (Hudson et al., 1983; Bathgate et al., 2013). All of the members subsequently discovered of the relaxin family retain the relaxin-like pre-prohormone structure and are predicted or proven to have the same processing and structure (Bathgate et al., 2013).

**Figure 2:**
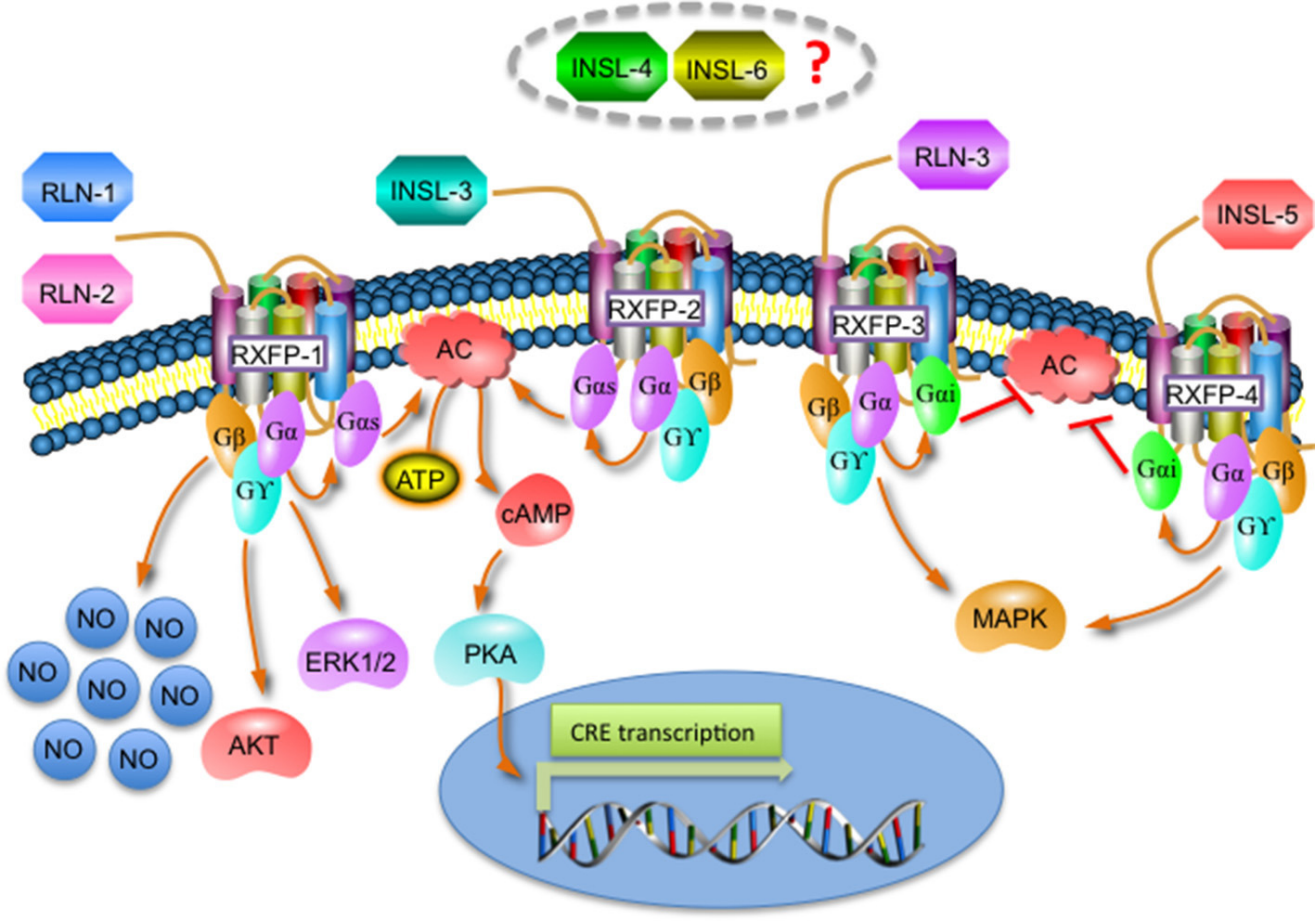
Overview of the different signaling pathways activated by the relaxin family and its receptors.

Although relaxin peptides are structurally related to insulin, they have low sequence similarity and bind to a different type of receptors. Relaxin peptides activate a group of four G protein-coupled receptors (GPCRs): the relaxin family peptide receptors (RXFP) 1-4; whereas insulin activates tyrosine kinase receptors (Wilkinson et al., 2005; Siddle, 2011; Bathgate et al., 2013). Relaxin 1/2 and INSL-3 bind to RXFP-1 and RXFP-2, respectively. RXFP-1 activation triggers signaling pathways mainly related to the generation of second messengers like nitric oxide (NO) or cyclic adenosine monophosphate (cAMP) (and the subsequent activation of protein kinase A (PKA) and the cAMP-response element (CRE)-mediated transcription), and also stimulates the phosphorylation of mitogen-activated protein (MAP) kinases like ERK1/2 or AKT, while RXFP-2 activation only induces cAMP and CRE-dependent gene transcription (Bathgate et al., 2013). RLN-3 and INSL-5 activate RXFP-3 and RXFP-4 respectively, which inhibit cAMP production and activate MAP kinases. The receptors for INSL-6 and INSL-4 remain currently unknown (Figure 2; Bathgate et al., 2013).

Different studies have shown a wide distribution of relaxin receptors in humans and murine, including ovary, prostate, brain, kidney, liver, pancreas, skeletal muscle, ligament, tendon, joint tissues, thymus, thyroid, adrenal glands, heart, arteries and veins (Halls et al., 2007; Clifton et al., 2014; Jelinic et al., 2014; Kim et al., 2016). Thus, this wide distribution of relaxin receptors in different species supports the potential pleiotropic effects of relaxin.

### Relaxin as a metabolic hormone

The similar structure of relaxin and insulin suggests that relaxin might have some important actions in regulating energy metabolism. In this line, it has been shown the expression of relaxin receptors in important organs for insulin action, like the pancreas, liver or muscle (Halls et al., 2007), and there exist some studies that propose relaxin as a new potential energy-regulating peptide. In fact, relaxin has been shown to promote glycogen depletion and to induce morphological changes of hepatocytes, which are consistent with functional activation, in both male and non-pregnant female rats (Bani et al., 2001b), and the peripheral infusion of relaxin in C57BL/6J mice has been shown to enhance insulin-stimulated muscle glucose uptake when animals are fed with normal diet but not when fed with high fat diet, and to reverse diet-induced insulin resistance in those fed with high fat diet, suggesting that relaxin can be an effective new molecule to revert muscle insulin resistance (Bonner et al., 2013). One of the mechanisms that trigger insulin resistance is the inflammation produced in obesity, due to the impairment of the adipose tissue homeostasis and the imbalance on the adipokine production toward a pro-inflammatory profile (Blüher, 2016). So far, little is known about the mechanisms through which relaxin seems to ameliorate insulin resistance and more studies are needed, but the control of the inflammatory processes linked to the development of impaired insulin sensitivity could be one of the involved pathways and is an interest field of study regarding the possible relaxin use as a therapy in CMDs.

Relaxin circulating concentrations in women with T2DM have been found to be negatively correlated to pancreatic β-cells activity, but positively correlated to insulin sensitivity and to other factors that are closely related to pancreatic function and insulin sensitivity, like fasting circulating concentrations of insulin, total cholesterol and LDL cholesterol, or C peptide, suggesting that relaxin may protect against insulin resistance in women with T2DM (Szepietowska et al., 2008). Serum relaxin concentrations have also been shown to be elevated in pregnant women with T1DM (Whittaker et al., 2003), in pregnant women with early gestational diabetes mellitus (Alonso Lopez et al., 2017), in non-pregnant women with metabolic syndrome (Ghattas et al., 2013). However, in a study composed by men and women, circulating relaxin concentrations were lower in patients with T2DM than in controls but not related to component traits in patients with diabetes such as cholesterol, triglycerides, fasting blood glucose or fasting insulin (Zhang et al., 2013). This difference could be explained due to the sample size and the gender differences between studies.

Despite de fact that (a) relaxin shares its structure with insulin, (b) it seems to improve insulin resistance, and (c) its circulating levels are altered in diabetes, it is not clear whether or not relaxin could share with insulin the capacity to decrease blood glucose levels. Although, in healthy C57BL/6J mice fed with high fat diet (Bonner et al., 2013) and in genetically diabetic *db*^+^*/db*^+^ mice (Bitto et al., 2013) the treatment with relaxin was shown to decrease fasting blood glucose levels (Bonner et al., 2013), there exist other studies with diabetic animal models in which relaxin do not normalize circulating glucose concentrations (Dschietzig et al., 2015; Ng et al., 2017) or glycosylated hemoglobin (Wong et al., 2013). Thus, more studies are needed to elucidate if relaxin could indeed mimic insulin and decrease circulating glucose levels. In regard with this aspect, even though insulin and relaxin activate a different type of receptors, both insulin receptors and RXFP-1 signaling can converge in the activation of AKT (Zaid et al., 2008; Bathgate et al., 2013; Sun et al., 2016; Ogunleye et al., 2017), which is a key kinase implicated in glucose transporter-4 (GLUT-4) mobilization to the cell membrane and glucose uptake in different tissues, contributing to the lowering of glucose circulating concentrations (Sakamoto and Holman, 2008). This observation suggests that relaxin could potentially participate in the regulation of blood glucose levels, but more studies are needed to clarify this issue.

Regarding food intake regulation, the neuropeptide RLN-3 is the one of the relaxin family that was by far more studied. Centrally administered RLN-3 in rats has been shown to increase water intake, food intake and body weight in males (McGowan et al., 2005; Hida et al., 2006; Otsubo et al., 2010). When comparing the different effect of RLN-3 central injection between sex, female rats show a higher increase in food intake and in body weight gain compared to males, and it induces a different corticotrophin-releasing hormone (CRH) expression pattern in different paraventricular hypothalamic nucleus (PVN) areas between male and female rats, an effect suggested to mediate the different response to RLN-3 on food intake behavior between sex (Lenglos et al., 2015). On the other hand, the intake of rewarding substances, like sucrose or alcohol, has been shown to increase endogenous RLN-3 levels in the brain (Ryan et al., 2014); and in rats with diet-induced obesity (DIO) it was shown that central RLN-3 expression is constitutively increased, and that refeeding after food deprivation stimulates the orexigenic effect of RLN-3 through the increase of RXFP-3 expression in brain areas that regulate food intake (Lenglos et al., 2014). The orexigenic effect of RLN-3 through its cognate receptor RXFP-3 has been proved to be mediated by the hyperpolarization and consequent inhibition of the majority of putative magnocellular PVN neurons, including cells producing the anorexigenic neuropeptides, oxytocin and vasopressin (Kania et al., 2017).

Although there exist more studies so far focused on the neuropeptide RLN-3 role regarding food intake behavior than on relaxin, these works could provide some clues and open a new line of study concerning the possible role of relaxin in food intake regulation and its concomitant effect on CMDs. In fact, and on the contrary of RLN-3, central and peripheral administration of RLN-2 in *ad libitum*-fed male rats has been shown to reduce food intake (McGowan et al., 2010).

Relaxin has been identified as a secreted factor in porcine adipose tissue (Hausman et al., 2006), and to induce hypertrophy in mammary and parametrial adipose tissue in female mice and in 3T3-L1 preadipocytes (Bianchi et al., 1986; Bani et al., 1989; Pawlina et al., 1989), and to promote lipid deposition in the parametrial adipose tissue in mice (Bani et al., 1989). Although the adipose tissue seems to be a target organ for relaxin, it is unknown whether or not the different adipose tissue depots express relaxin receptors, and whether or not relaxin could participate in the regulation of adipose tissue homeostasis in terms of growth, energy metabolism or even adipokine secretion, with the concomitant effect on systemic inflammation and the development of CMDs.

### Relaxin and relaxin receptors expression in the cardiovascular system

Relaxin and its receptors are widely located in different cardiovascular tissues. It has been demonstrated the expression of RXFP-1 in rodents in the aorta, vena cava, mesenteric artery, mesenteric vein, femoral artery, femoral vein, small pulmonary arteries and small renal arteries (Novak et al., 2006; Jelinic et al., 2014), as well as in cardiomyocytes (Moore et al., 2009) and in cardiac atrial and left ventricle tissue, with higher expression in atria (Osheroff and Ho, 1993; Hsu et al., 2000; Kompa et al., 2002; Krajnc-Franken et al., 2004; Scott et al., 2004). Moreover, relaxin shows a specific and high-affinity binding to its receptors in the atrium in both male and female rat heart (Osheroff et al., 1992). RXFP-1 has also been detected in human heart (Hsu et al., 2002; Dschietzig et al., 2011), again with higher expression in atria, and its expression is enhanced by α1-adreonreceptors stimulation but suppressed by β1-adrenoreceptors activation in cultured rat cardiomyocytes and in transgenic mouse hearts with cardiac-restricted overexpression of subtypes of adrenoceptors (Moore et al., 2009, 2014).

Likewise relaxin receptors, relaxin is also expressed in cardiovascular tissues. In rodents, relaxin has been detected in thoracic aortas, mesenteric arteries, small renal arteries, in rat heart tissue and in cultured cardiomyocytes derived from the atria of neonatal rats, which secrete relaxin in detectable amounts (Taylor and Clark, 1994; Gunnersen et al., 1995; Novak et al., 2006), and RLX-3 is also detected in the atria and ventricle in mice and rats (Bathgate et al., 2001; Kompa et al., 2002). In humans, relaxin was demonstrated to be expressed in atrial and ventricular cardiac tissue (Dschietzig et al., 2001).

### Relaxin effects at cardiovascular level

Relaxin has been demonstrated to participate in the cardiovascular and hemodynamic changes required to adapt the cardiovascular system to pregnancy, so that during pregnancy take place increases in plasma volume, cardiac output or heart rate, and decreases in blood pressure and vascular resistance (Bathgate et al., 2013). However, relaxin can also regulate cardiovascular function in men and non-pregnant women at different levels, modulating blood pressure, inflammation, cell injury/death, fibrosis, hypertrophy or angiogenesis (Teichman et al., 2010; Leo et al., 2016a).

#### Relaxin effects on vasculogenesis and vascular function

Relaxin is able to stimulate the formation of new blood vessels, not only in pregnancy but also in tumorigenesis or ischemic wounds, through the upregulation of vascular endothelial growth factor (VEGF) transcripts (Shirota et al., 2005; Silvertown et al., 2006; Segal et al., 2012; Bitto et al., 2013; Unemori et al.). And in genetically diabetic mice, relaxin not only increases new vessel formation but also improves the impaired wound healing, suggesting that it could be beneficial in diabetes-related wound disorders (Squadrito et al., 2013).

The endothelial cells are the key regulators of the vascular tone through the production and secretion of vasoactive substances, including vasodilator factors such as NO, prostacyclin (PGI_2_), kinins (bradykinin), or endothelium-derived hyperpolarizing factors (like K^+^ ions), and vasoconstrictor agents such as endothelin-1, thromboxane A or angiotensin II (AngII) (Félétou and Vanhoutte, 2006; Su, 2006).

A big number of studies have shown that relaxin promotes vasodilation through a mechanism that involves NO production in a wide range of organs/tissues, not only in reproductive organs such as the mammary glands (Bani et al., 1988) or the uterus (Vasilenko et al., 1986; Bani et al., 1995), but also in non-reproductive tissues like the mesocaecum (Bigazzi et al., 1986), kidney (Danielson et al., 1999, 2000; Novak et al., 2001; Conrad et al., 2004; McGuane et al., 2011), subcutaneous fat (McGuane et al., 2011) or liver (Bani et al., 2001a).

In the heart, relaxin increases coronary flow in normal and hypertensive rats (Bani-Sacchi et al., 1995; Masini et al., 1997; Debrah et al., 2005b). Relaxin has also been shown to decrease systemic arterial resistance and to increase global artery compliance in rats (Conrad et al., 2004; Debrah et al., 2005a,c, 2006; Conrad and Shroff, 2011), as well as it reverses large artery remodeling and improves arterial compliance in senescent spontaneously hypertensive rats (Xu et al., 2010). In pregnant relaxin-deficient mice, relaxin administration for 5 days has been shown to prevent vascular dysfunction in mesenteric arteries and to ameliorate the increased responsiveness of small mesenteric arteries to the vasoconstrictor AngII, suggesting that relaxin could alleviate maternal systemic vascular dysfunction associated with hypertensive diseases in pregnant women (Marshall et al., 2017).

Recombinant human relaxin in co-treatment with high doses of glucose for 3 days was also demonstrated to prevent vascular dysfunction in the mouse aorta through a mechanism that reverts the reduced sensitivity to the endothelium-dependent agonist acetylcholine induced by high glucose, and that ameliorates PGI_2_ production (Ng et al., 2016). In streptozotocin induced diabetic mice, relaxin treatment for 2 weeks reversed diabetes-induced endothelial dysfunction in terms of endothelial vasodilator function in mesenteric arteries and aorta by increasing NO and PGI_2_ mediated relaxation, but it did not affect endothelium-derived hyperpolarizing factors (Ng et al., 2017).

Acute infusion of relaxin (3 h) in healthy male rats has also been shown to increase in the mesenteric artery basal NOS activity and to reduce endothelin-1 dependent contraction, and this vasodilator effect was sustained for 24 h due to the following increase in PGI_2_/bradykinin production, even though the absence of circulating levels of relaxin at 24 h (Leo et al., 2014). Similarly, in male rats continuously infused with relaxin, it was shown an increase in the endothelial vasodilator function in arteries, but not in veins, through the production of NO and the increase of eNOS activity at 48 h, a mechanism reverted at 72 h, but at this time, relaxin induced a transition to PGI_2_ and bradykinin production, a mechanism suggested by the authors to be key to sustain vascular response to relaxin in time (Leo et al., 2016b). The same effects are observed when relaxin is administered chronically (5 days) in male rats: relaxin reduces wall stiffness and increases volume compliance in mesenteric arteries through the increase of bradykinin-mediated relaxation, involving enhanced NO production but not endothelium-derived K^+^ hyperpolarization, and in this study PGI_2_ production was not observed (Jelinic et al., 2014). On the other hand, in blood-perfused hamster cremaster muscle preparations *in situ*, relaxin induced a rapid (seconds), transient vasodilation in transverse and branch arterioles through NO production and K^+^ hyperpolarization, while the smallest ramification of the arteriolar tree was not responsive to relaxin (Willcox et al., 2013). However, it was also shown that 48 h intravenous relaxin infusion in healthy rats does not significantly alter resting outer diameter or pressure-induced myogenic tone in the mesenteric vasculature despite enhancing the contribution of NO through increased endothelial NO synthase (eNOS) dimerization (Jelinic et al., 2017).

Taken these results all together, it seems clear that relaxin has a potent vasodilator effect, and that contributes to ameliorate endothelial dysfunction in cardiometabolic scenarios such as hypertension or diabetes. It was recently suggested that endothelial cells have functional heterogeneity depending on the tissue, being determined by mechanical and metabolic stimuli, as well as by the characteristic microenvironment of each tissue (Potente and Mäkinen, 2017), and also between sex (Mudrovcic et al., 2017). Thus, the differences observed regarding timing and the specific pathways activated by relaxin in the different studies could be due not only to the different experimental designs and animal models used or relaxin doses, but also to a different response by endothelial cells from different tissues/physiopathological conditions to relaxin.

Apart from the regulation of the vascular tone, endothelial cells mediate other functions, such as the preservation of blood fluidity, the formation of new blood vessels, platelet function, vascular smooth muscle cell growth and migration or the regulation of the inflammatory response (Jensen and Mehta, 2016; Incalza et al., 2017). Under pathological scenarios associated with a pro-inflammatory profile, such as obesity, diabetes, hypertension or dyslipidemia, the endothelial cells are influenced by cytokines and external stimuli to change into a pro-inflammatory and pro-coagulant state, characterized by the expression of cell-surface adhesion molecules required for the recruitment and attachment of inflammatory cells, which lead to clot generation, increasing the thrombotic risk as a consequence of increased blood thrombogenicity or impaired fibrinolysis (Incalza et al., 2017; Montecucco et al., 2017). Thus, and although little is known so far, the proved effect of relaxin on regulating endothelial function suggests that relaxin could also ameliorate the inflammatory response in the vascular system under pathological conditions, and this opens a promising new field of study of relaxin regarding its potential role as a regulator of cardiovascular inflammation. In fact, in human endothelium and vascular smooth muscle cells, relaxin was already proved as a potent inhibitor of early vascular inflammation, decreasing the expression of endothelial adhesion molecules, cytokine expression and suppressing monocyte adhesion to the endothelium (Brecht et al., 2011), a result also observed *in vivo* in female apolipoprotein E-deficient mice fed with a high-fat and cholesterol-rich diet for 6 weeks, in which relaxin treatment for the last 4 weeks reduced vascular oxidative stress, improved endothelium-dependent vasodilatation, reduced the development of the atherosclerotic plaque, decreased circulating concentrations of the cytokines interleukin (IL)-6 and IL-10, and down-regulated the angiotensin II type 1a receptor in the aorta, but in this study authors did not find differences in vascular macrophage, T-cell or neutrophil infiltration, nor in collagen/vascular smooth muscle cell content between relaxin treated and control mice (Tiyerili et al., 2016).

#### Chronotropic and inotropic effects of relaxin in the heart

In the heart, relaxin has powerful positive chronotropic and inotropic effects. It has been shown to induce an increase in the contraction force and rate in isolated rat atria, and in conscious normotensive and spontaneously hypertensive rats relaxin increases heart rate without alter urine or blood volume, mean arterial pressure of water and food intake (Kakouris et al., 1992; Ward et al., 1992; Toth et al., 1996). In rat perfused hearts, relaxin infusion has been shown to induce the release of the atrial natriuretic peptide (ANP) along with the increase in heart rate through a mechanism that involves protein kinase C (PKC) activation (Toth et al., 1996), and in isolated murine cardiac myofilaments relaxin increases cardiac myofilaments force through a PKC-dependent pathway that leads to the increase of myofilament Ca^2+^ sensitivity (Shaw et al., 2009). As well, in rat isolated hearts it causes a dose-dependent tachycardia in both intact preparations and those in which the atria had been removed, suggesting that relaxin acts on both the atrial and ventricular pacemakers to increase the heart rate (Thomas and Vandlen, 1993). In fact, it was demonstrated in single cells isolated from the sinoatrial node in rabbits that relaxin is able to enhance L-type Ca^2+^ current through a mechanism dependent on cAMP formation and PKA activity (Han et al., 1994). In human myocardium, relaxin has positive inotropic effects in atrial tissue, without differences between control and failing hearts, through a mechanism that involves PKA activation and a decrease in the transient K^+^ outward current, an effect partially blunted by the pretreatment with pertussis toxin and the inhibition of phosphoinositide-3 kinase (PI3K) in non-failing hearts but notably suppressed in failing myocardium (Dschietzig et al., 2011). However, in this study, relaxin did not show any inotropic effects in ventricular myocardium.

#### Relaxin and ischemia-reperfusion injury

Relaxin has been extensively proven to protect the heart against damage induced by ischemia/reperfusion. The process of ischemia/reperfusion induces the generation of O_2_-derived free radicals, that contribute to the peroxidation of cell membrane lipids and to damage the mitochondrial function, and the overload of Ca^2+^, which alters myofilaments contractile function and triggers proteolytic cascades, leading to cell injury (Anderson et al., 2012; Bompotis et al., 2016). In isolated guinea pig heart, relaxin was shown to protect myocardium from ischemia/reperfusion injury by decreasing the peroxidation of cell membrane lipids and Ca^2+^ overload, as well as the hypercontraction of myofibrils, mitochondrial swelling and accumulation of dense granules in the mitochondrial matrix, through a mechanism that involves NO production (Masini et al., 1997).

As well, in a swine model of acute myocardial infarction, relaxin injection during reperfusion caused a reduction in circulating markers of myocardial injury, as troponin T, creatin kinase-MB or myoglobin, and in tissue malondialdehyde (an end product of lipid peroxidation) and Ca^2+^ (mediate cardiomyocyte injury), caspase-3 (implicated in cardiomyocyte apoptosis), and myeloperoxidase (which recruits inflammatory leukocytes), and improved cardiac contractile function (Perna et al., 2005). According to this, other authors have found that relaxin also protects from the damage induced by ischemia and reperfusion in rat heart by a similar mechanism, so that intravenous relaxin injection 30 min before ischemia diminished the extension of the damaged areas, ventricular arrhythmias, the recruitment and accumulation of neutrophils and morphological signs of myocardial cell injury, by decreasing oxygen-derived free radicals, preventing the Ca^2+^ overload in the myocardial tissue, and reducing hypercontraction of myofibrils, mitochondrial calcification, and cell necrosis (Bani et al., 1998). Moreover, in rats with isoproterenol-induced myocardial injury, it was found a compensatory up-regulation of myocardial relaxin expression, and when relaxin was co-administered with isoproterenol for 10 days, it attenuated myocardial injury and fibrosis, and improved cardiac function (Zhang et al., 2005). In this line, in a rat model with myocardial infarction, subcutaneously administrated relaxin during 2 weeks was probed to attenuate tachyarrhythmia and cardiac dysfunction in the healing infarcted heart, to reduce the dispersion of action potential duration in post-infarcted hearts, to reduce myocardial apoptosis and cardiac fibrotic collagen deposition and to inhibit protein expression levels of tumor growth factor (TGF) β1, α-SMA, and type I collagen (Wang et al., 2016). In a different murine model of myocardial infarction, it has been shown that relaxin administration (1 h prior to ischemia or as a reperfusion therapy) attenuates myocardial ischemia/reperfusion injury by reducing infarct size and left ventricular dysfunction after 24 h through a mechanism that involves eNOS signaling and the attenuation of the activation of the Nod like receptor containing a pyrin domain-3 (NLRP3)-inflammasome (Valle Raleigh et al., 2017), which is a macromolecular structure that functions as a platform for the production of pro-inflammatory cytokines of the IL-1 family (i.e., IL-1b and IL-18) and is involved in the impairment of heart function and remodeling after myocardial injury (Mezzaroma et al., 2011; Lamkanfi and Dixit, 2012; Bracey et al., 2013).

After the induction of myocardial infarction in swine and rats, the transplantation of skeletal myoblasts overexpressing relaxin was probed as an effective treatment to increase vascularization, increase collagen turnover, reduce fibrosis, and improve left ventricular function, compared to non-overexpressing skeletal myoblast (Formigli et al., 2007; Bonacchi et al., 2009).

#### Relaxin and atrial fibrillation

Atrial fibrillation (AF), defined as a supraventricular tachyarrhythmia due to uncoordinated atrial activation with deterioration of the atrial mechanical function, is nowadays one of the cardiovascular events that are causing an extremely costly public health problem, being sex, age and hypertension the main risk factors for its development (Fuster et al., 2006). Thus, the understanding of the mechanisms related to AF development/prevention is of a great interest.

In spontaneously hypertensive rats, relaxin treatment for 14 days was shown to suppresses AF through the inhibition of fibrosis and hypertrophy, and the increase in conduction velocity, and in human cardiomyocytes derived from inducible pluripotent stem cells, relaxin treatment for 48 h was probed to up-regulate voltage-gated Na^+^ channels, a mechanism suggested by the authors to participate in the suppression of AF (Parikh et al., 2013), a result also observed in aged rats (Henry et al., 2015). As well, in mice with myocardial infarction, relaxin treatment after myocardial infarction for 14 days also reduces AF through the decrease on fibrosis and hypertrophy, the increase in conduction velocity, and, moreover, the decrease of the pro-inflammatory cytokine IL-1β expression (Beiert et al., 2017).

In humans, circulating relaxin was found to be increased in patients with AF and to be associated with serum concentration of fibrosis-related markers, as well as with the occurrence of heart failure in AF patients (Zhou et al., 2016).

#### Relaxin effects on cardiac cells

Apart from the numerous studies regarding relaxin effects on the cardiovascular system and in heart physiology, there are also some reports concerning relaxin direct effects on cardiomyocytes and cardiac fibroblasts.

In neonatal rat atrial and ventricular fibroblasts in culture, relaxin was shown to decrease collagen secretion and deposition by the inhibition of fibroblasts proliferation and differentiation, and the enhancement of matrix metalloproteinase activity, an effect also observed in two models of cardiac fibrosis *in vivo*, in which relaxin is able to revert collagen overexpression (Samuel et al., 2004; Mookerjee et al., 2005; Wang et al., 2009). In cardiac fibroblasts, relaxin co-treatment with high glucose was suggested to inhibit high glucose-associated cardiac fibrosis partly through the decrease in total expression and translocation of PKCβ2 (Su et al., 2014).

In mouse neonatal immature cardiomyocytes, relaxin promotes cell proliferation and maturation (Nistri et al., 2012), an effect that is also potentiated when are co-cultured with relaxin overexpressing skeletal myoblasts (Formigli et al., 2009). In fact, relaxin has been shown to potentiate intercellular coupling between myoblasts and cardiomyocytes by up-regulating the transcellular exchange of regulatory molecules between both cell types (Formigli et al., 2005).

Relaxin was demonstrated to inhibit the ability of cardiac fibroblast-conditioned medium to induce hypertrophy in cardiomyocytes, and to directly attenuate apoptosis induced by oxidative stress and by high glucose exposure in cardiomyocytes through a protective mechanism that involves AKT and ERK activation (Moore et al., 2007), and through the inhibition of both extrinsic and intrinsic pathways of apoptosis and endoplasmic reticulum stress (Zhang et al., 2015).

## Relaxin as a future therapy for cardiometabolic diseases: lights and shadows

**Figure 3:**
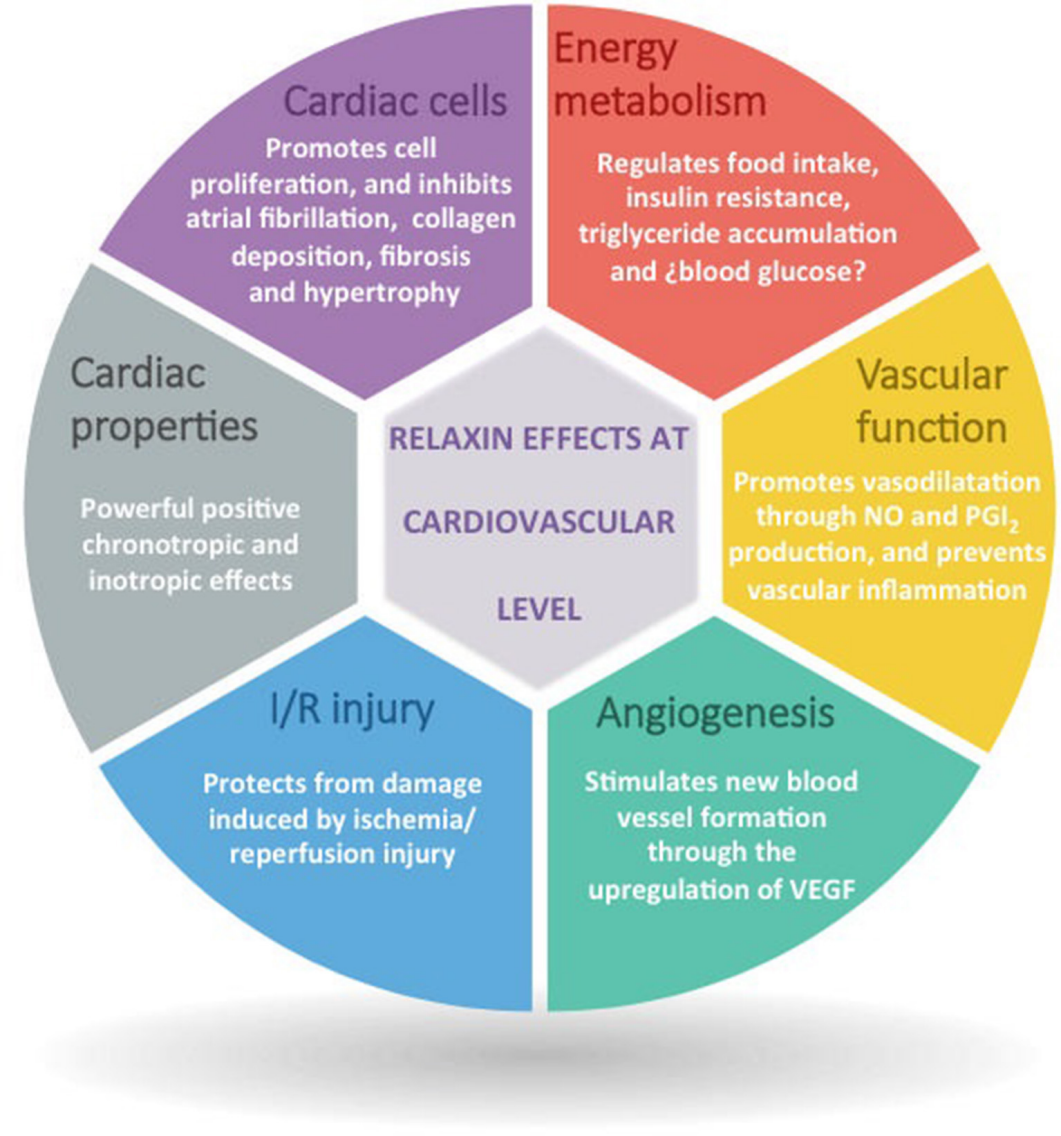
Overview of the main relaxin actions at cardiovascular level.

Due to the important relaxin effects not only on the cardiovascular system but also in the development of metabolic disorders that suppose risk factors for the development of cardiovascular diseases (Figure 3), relaxin has been considered in the last years as a really promising cardiometabolic hormone that with its therapeutic modulation could help to prevent/treat cardiovascular diseases. Although relaxin has been probed to participate in the pathophysiological processes that lead to CMDs, is just in the scenario of acute heart failure where relaxin has created interest as a therapeutic agent. In this line, human recombinant relaxin (serelaxin/RLX030) has been under commercial development by Novartis Pharma A.G. (Basel, CHE) and it was first tested in healthy or hypertensive rodents and humans, proving its capacity to increase systemic vasodilatation, global arterial compliance, cardiac index and stroke volume, and to decrease arterial stiffness (Du et al., 2014). Subsequently, its safety, tolerability and beneficial effect was tested in phase I and II clinical trials in stable and acute heart failure patients (Dschietzig et al., 2009; Teerlink et al., 2009; Sato et al., 2015), and in 2013 there were published the results of the phase-III multicenter, randomized and placebo-controlled (RELAX-AHF) trial (Teerlink et al., 2013), consisted in 1,161 acute heart failure patients; 581 patients treated with serelaxin and 580 patients receiving placebo, showing that the infusion of serelaxin for 48 h improved dyspnea, and reduced heart failure events, congestion, the length of hospital stay and the intensive care, as well as it reduced cardiovascular and all-cause mortality, blood pressure, and renal adverse events compared with placebo, independently of having preserved or reduced left ventricle ejection fraction (Teerlink et al., 2013; Filippatos et al., 2014).

Despite these encouraging results, a real-world patients (5,856) study designed to further analyze the RELAX-AHF results showed that only 23% of all consecutive patients hospitalized with acute heart failure met criteria of the RELAX-AHF trial, and that the mortality rates were lower in participants of ongoing randomized clinical trials in comparison with real-world acute heart failure patients (Spinar et al., 2017).

Recently, it has been developed the RELAX-AHF-2 study to corroborate the promising results of serelaxin observed in the RELAX-AHF. RELAX-AHF-2 is a multicenter, randomized, double-blind, placebo-controlled, event-driven, phase III trial involving ~6,800 patients hospitalized for acute heart failure with persistent dyspnea and pulmonary congestion, elevated natriuretic peptide levels, mild-to-moderate renal impairment, and systolic blood pressure ≥125 mmHg. The primary objectives of this study are to probe that serelaxin is superior to placebo in decreasing 180 days cardiovascular death, and the reduction of occurrence of worsening heart failure through day 5. Key secondary endpoints include 180 day all-cause mortality, composite of 180 day cardiovascular death or rehospitalization due to heart/renal failure, and in-hospital length of stay during index acute heart failure (Teerlink et al., 2017). Although the results from this study have not been published yet, Novartis has recently provided a report announcing that the RELAX-AHF-2 do not confirm the efficacy of serelaxin in acute heart failure, so that it does not meet its primary endpoints of reduction in cardiovascular death through day 180 or reduced worsening heart failure through day 5 (Novartis provides update on Phase III study of RLX030 (serelaxin) in patients with acute heart failure | Novartis)^1^. Thus, the real effect of serelaxin as an improver of heart failure should be deeply studied.

## Conclusion

Relaxin is a cardiometabolic hormone with important impact on the cardiovascular pathophysiology. Although relaxin beneficial effects on acute heart failure patients have been previously proved, nowadays its beneficial effect is under controversy due to the contradictory results found between the RELAX-AHF and both the RELAX-AHF-2 and a real-world patients study. Moreover, the precise mechanism through which relaxin act in different CMDs is not known yet, neither the mechanisms that regulate relaxin and its receptor expression in the different tissues in which they are produced. Furthermore, it was also suggested that different concentrations of relaxin can activate its receptor in a different way (Bathgate et al., 2013), so that the regulation of relaxin effects in different tissues depending on its concentration could be difficult to comprehend. Overall, it seems clear that relaxin is a new potential candidate as a therapeutic agent to treat/prevent cardiometabolic diseases, so that it has clear effects on vascular function, has positive chronotropic and inotropic effects in the heart, and prevents ischemia/reperfusion injury and atrial fibrillation. Although, further studies are needed, it also seems to be a potential regulator of metabolism, so it could regulate the metabolic disturbances observed in CVDs.

## Author contributions

SF, JG, and FL: Manuscript redaction and revision. AA, DR, MP, ER, and MR: Manuscript redaction.

### Conflict of interest statement

The authors declare that the research was conducted in the absence of any commercial or financial relationships that could be construed as a potential conflict of interest.

## Footnotes

^1^Novartis provides update on Phase III study of RLX030 (serelaxin) in patients with acute heart failure | Novartis Available online at: https://www.novartis.com/news/media-releases/novartis-provides-update-phase-iii-study-rlx030-serelaxin-patients-acute-heart [Accessed May 4, 2017].
